# Strategies to reduce inappropriate admissions to the neurologic ward: a model of hospital-territory interaction in the management of acute episodes

**DOI:** 10.3389/fpubh.2023.1188592

**Published:** 2023-06-30

**Authors:** Maurizio Giorelli, Pasquale Di Fazio

**Affiliations:** ^1^Operative Unit of Neurology, “Dimiccoli” General Hospital, Barletta, Italy; ^2^Operative Unit of Nuclear Medicine, “Dimiccoli” General Hospital, Barletta, Italy

**Keywords:** inappropriate admissions, neurological disorders, emergency department, sustainability, management of emergency events, cost/effectiveness

## The economic sustainability of public health and the burden of inappropriate admissions

A major challenge faced by modern public health systems is the provision of affordable high-quality medical care. The availability of expensive pharmacological treatments and medical devices has pushed the economic boundaries of healthcare, making the management of limited economic resources critical (1). From an organizational point of view, hospital admissions are defined as inappropriate when clinical cases could be addressed at a less intensive level of care, with equal effectiveness and greater economic use of resources. Furthermore, hospital admission of fragile and comorbid patients worsens their quality of life, possibly resulting in nosocomial infections (2). Appropriateness has been identified as the “next frontier” in clinical practice development: a scenario in which doctors act according to established clinical-organizational guidelines, with economic reimbursement advantages, while respecting individual patient needs.

## The Italian law

Inappropriate hospital admissions arise from diagnosis-related groups (DRGs) of low complexity that could be managed by outpatient services with the same clinical outcomes and lower resource consumption according to “Decreto del Presidente del Consiglio dei Ministri” of Novembre 29th, 2001.^1^ The law cites 43 DRGs in the Level of Essential Assistance (LEA), which should be provided at a medical assistance level lower than that provided by hospitalization. The transfer of cases managed in the traditional regime, toward less intensive and less costly assistance is influenced by either clinical (severity, presence of other diseases, and level of self-sufficiency) (1) or sociodemographic aspects (economic condition of the family, presence of caregiver support, advanced age, and level of education) (3, 4).

## Inappropriate admissions to the neurologic ward of patients accessing the emergency department: the local situation

Avoiding inappropriate admission of patients to the neurologic ward from the emergency department (ED) is often complex (5, 6). A sudden exacerbation of chronic conditions or new episodes of known diseases may not necessarily require hospitalization, but rather short-term observation and management (7, 8). Chronic conditions that might exacerbate suddenly include the occurrence of single convulsive episodes in people with epilepsy, drug-resistant headaches in patients suffering from migraines, dizziness, behavioral and psychiatric symptoms, dementia (BPSD), and transient loss of consciousness associated with situational syncope. Individuals experiencing one of these pathological conditions are obliged to consult emergency services (ES) to address their needs (7–9). The ES, in turn, often has the primary task, under the current organization of healthcare systems in many regions of Italy and worldwide, to transfer the patient to a hospital-associated ED to ensure first aid and assessment. Additionally, many patients present to the ED expecting shorter waiting times than for outpatient visits.

**Figure 1:**
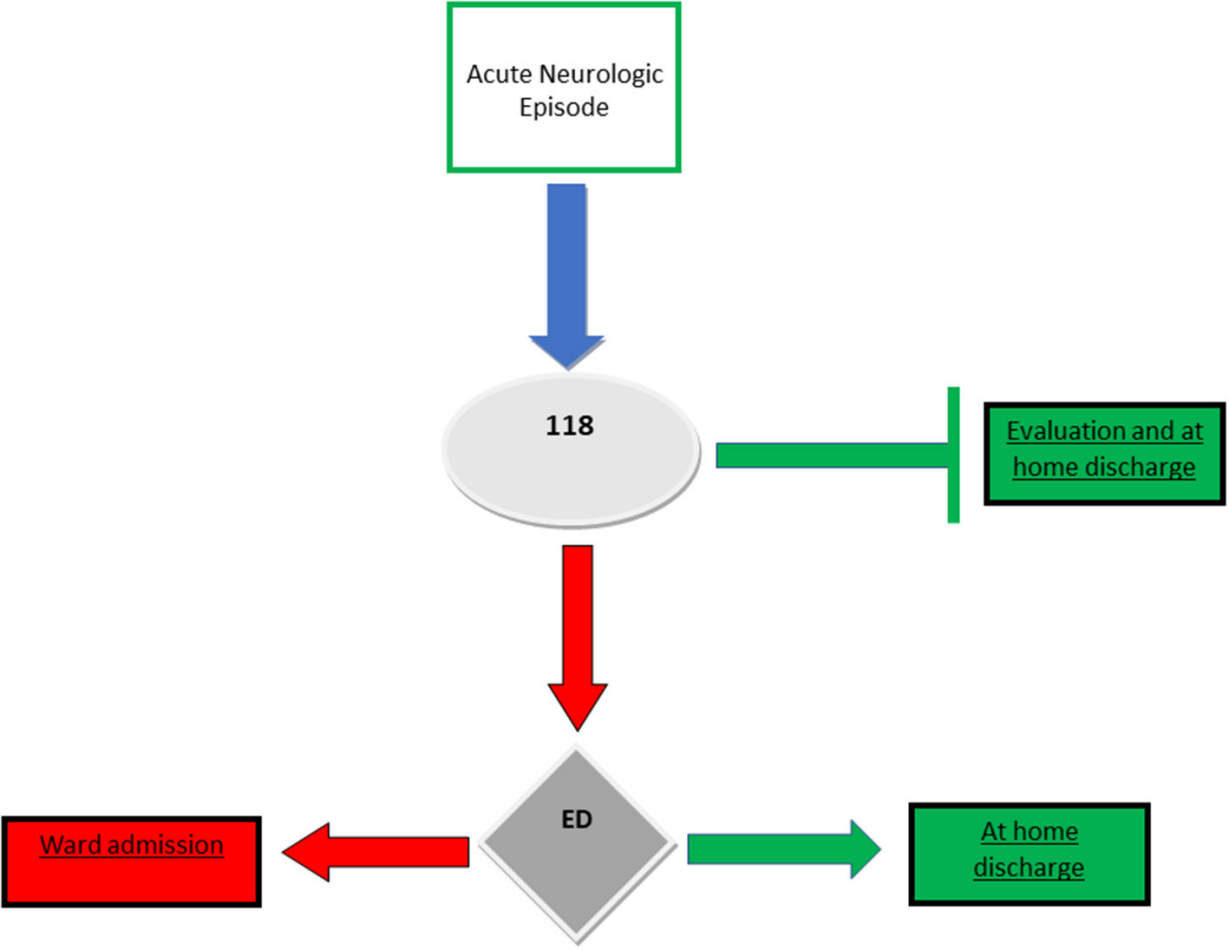
Former organization for management of emergency episodes at our local healthcare services (up to February 2023).

The “Dimiccoli” Hospital in Barletta serves a population of more than 200,000, within the Local Health Agency BT (LHA BT): Azienda Sanitaria Locale BT, ASL BT. The ED of this hospital manages >19,000 episodes/year, and the Operative Unit of Neurology manages >600 inpatients annually, with an average of 20% of inappropriate admissions arising from ED transfer in 2021. Prior to February 2023, the reason for this imperfect system was the lack of ED-associated structures devoted to short-term monitoring of patients under transitory unstable conditions but with favorable prognosis (Figure 1).

## Neurological episodes accessing the ED; giving rise to inappropriate admissions to the ward

Consistent with other countries, patients demonstrating epileptic episodes represent the largest number of inappropriate admissions in our neurological ward (10). These patients often present with signs that encourage hospital admission, such as somnolence, slight electrolyte imbalance, and abnormal cranial computed tomography (CT) or electroencephalogram (EEG) findings. Dissecting these factors and making decisions regarding at-home management is complex, even for highly skilled and experienced specialists. In most circumstances, careful evaluation, including collection of clinical history, neurological examination, complete blood examinations (including alcohol and abuse substances), brain CT, and EEG, might suffice to rule out red flags suspicious for symptomatic fits (11). Similarly, transient loss of consciousness, particularly if witnessed and preceded by prodromal symptoms, such as dizziness, paleness, diaphoresis, reduced visual acuity, or tinnitus, might clearly point toward a benign situational syncope if it occurs under predisposing conditions. Dizziness may arise from drug-related side effects, benign infections of the upper airways, or orthostatic hypotension, possibly due to dehydration or changes in antihypertensive therapy. A simple neurological evaluation, even without brain CT, could shed light on the correct etiology. A history of migraine with the same clinical characteristics as the episode under evaluation as well as the absence of a thunderclap onset of headache might rule out sentinel or symptomatic headache (12). The onset of BPSD, although worrying for relatives and physicians, requires evaluation of body temperature, gut canalization, aches, and hydration in patients affected by major neurocognitive disorders. Once symptomatic BPSD related to other clinical emergencies is ruled out, the symptoms can be treated in a comfortable domestic environment under medical surveillance (13). The sole opportunity for clinical evaluation, emotional pressure from the patient's kin, and medico-legal risks often prompts the specialist to approve admission to the neurological ward, albeit the patient could be managed in an outpatient service. However, the unavailability of a 24-h on-site neurologist or of facilities for short monitoring leaves ED physicians with the choice to transfer the patient to the ward or to take manage possible medicolegal issues (8, 9). All admissions become management concerns once the patients are discharged, and an administrative reimbursement sheet [Scheda di Dimissione Ospedaliera (SDO)] has been validated. In the face of a small clinical advantage, which could easily be achieved by transferring the patient to a lower-level healthcare facility, high economic expense and a poor cost-effectiveness is observed. However, identification of inappropriate DRGs with unfavorable cost-effectiveness results from a “*post-hoc* analysis” upon patient discharge, and every effort should be made to prevent such in-hospital admissions, through the establishment of a secure multi-level healthcare service network.

## Facilities which might relieve inappropriate admissions

Brief intensive observation (BIO) is an emergency management method for patients affected by acute clinical concerns that might resolve to complete recovery but require close monitoring (14). This modality, involves a high level of assistance due to the considerable commitment of medical and nursing staff, execution of diagnostic tests, clinical monitoring, and planning of therapeutic strategies, is provided in a defined and limited period (usually 24 h) to identify the most suitable level of assistance for that particular patient. Although BIO has been conceived for inclusion in EDs in Italy, not all EDs can rely on such facilities.

Day Service is aimed at the management of clinical cases, the solution of which requires multiple and multidisciplinary clinical and instrumental investigations, even complex, provided by a specific diagnostic therapeutic path centered on the patient's clinical problems. Day Service is a care model aimed at rationalizing hospital care by improving the appropriateness of hospital admission, allowing the transfer of a substantial share of activity from the inpatient regime to an outpatient model of alternative care.

The Community Hospital (CB) is aimed at patients who, following a minor acute episode or an exacerbation of chronic pathologies, require low-intensity clinical interventions, with potential delivery at home, but who are hospitalized in these structures in the absence of a suitable domicile and need continuous nursing assistance/health surveillance that cannot be provided at home.^2^

Admission to the CB is provided to patients requiring completion of the clinical stabilization process, a prognosis with a short-term resolution (within 30 days), coming from home or other residential facilities, from the ED, or being discharged from acute care hospitals.

Community Houses (CH) are further reduced forms of assistance that do not provide formal in-bed hospitalization but ensure continual (up to 24 h) assistance through general practitioners, specialized physicians, nurses, health technology, and Integrated Domiciliary Assistance (IDA).

## The need for integration and telemedicine

To date, the concerning inappropriate ward admissions and effective and timely management of patients' healthcare needs have remained unsolved. This failure is mainly due to the lack of integration among the involved structures. The National Recovery and Resilience Plan (NRRP) aims to integrate hospital services, local health services and social services. Therefore, the territorial interaction of the emergency service with the hospital, in managing exacerbated chronic conditions, such as the recurrence of epileptic episodes, resistant migraine crisis, or the onset of psychomotor agitation in people with dementia, remains fundamentally relevant. The availability of a telemedicine network may assist the connection of different healthcare levels in a vertical or horizontal manner, with patient needs at the center (15).

## Discussion

**Figure 2:**
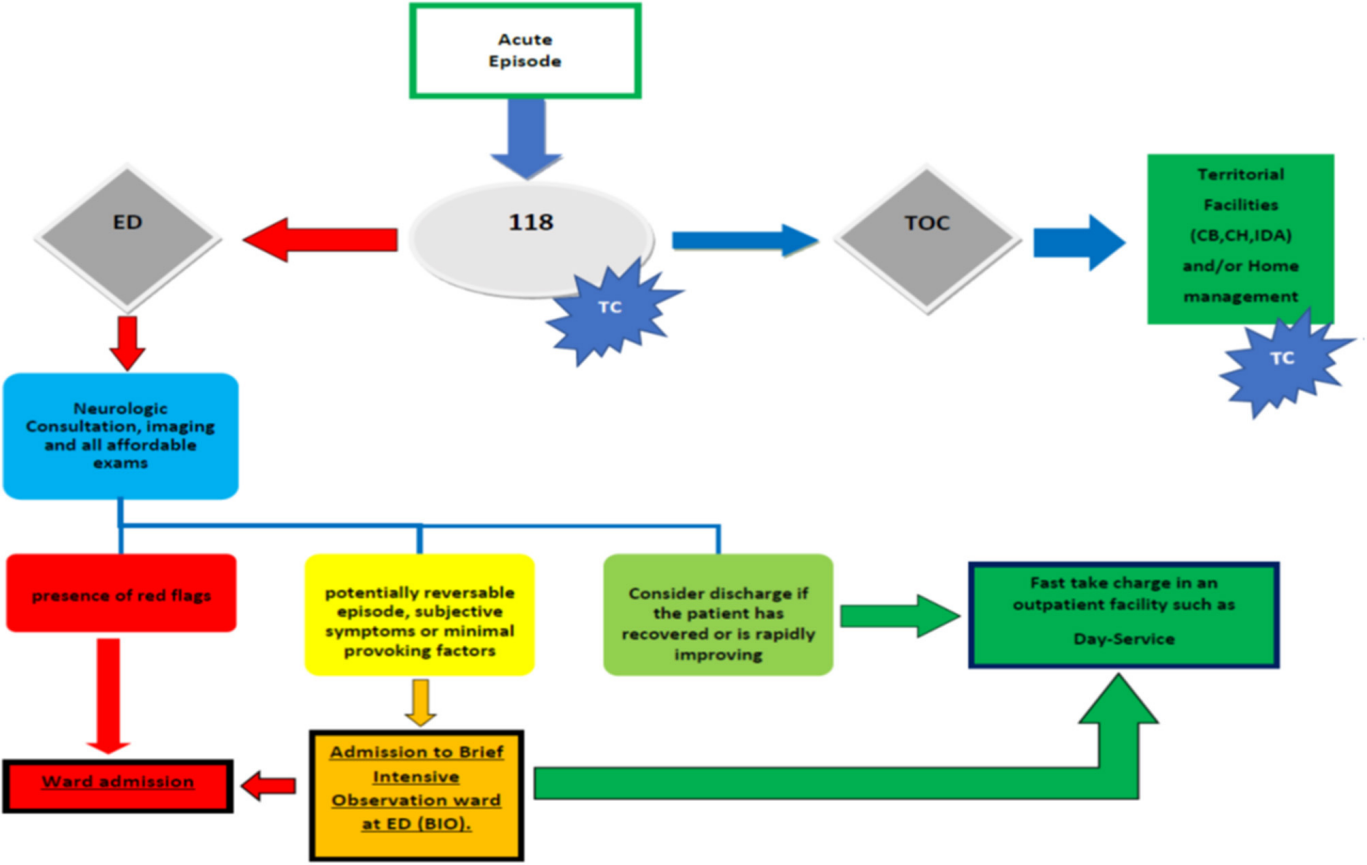
Putative network for management of neurologic emergencies; Hospital-territory interaction. ED, Emergency Department; TOC, Territorial Organizational Committee; 118, Emergency Medical Service; TC, Tele Consultation.

We propose a model that aims to manage neurological emergencies while reducing inappropriate admissions and improving the effectiveness of patient management by the National Health System (NHS) structures (Figure 2). However, interactions among facilities located in the territory [Territorial Organization Committee (TOC), CB, CH, and IDA] and within the hospital, are fundamental to the success of such a model. When a neurologic emergency arises, the 118 system, a facility to manage medical emergencies at home and evaluate specific patient needs, should interface with a neurologist through teleconsultation. Following specialist evaluation, if the final diagnosis confirms that the episode was derived from acute exacerbation of a chronic condition and that the patient's life and health risks are not severe, the patient should be directed by the TOC toward a territorial facility for either a brief admission in a low-level CB or home hospitalization under the supervision of IDA (Figure 2). Simple “home discharge” is possible for events that do not present clinical red flags and have complete restitution to previous condition. Neurological teleconsultation should be available at all territorial facilities to track the patients' conditions. The plasticity and reversibility of such a model are fundamental to ensure successful patient management.

Patients experiencing new symptoms, suspicious of an acute disease, unstable conditions, or possible clinical progression, should be referred to the ED for a complete evaluation. Based on the neurological consultation, examinations, and clinical outcomes, the patient could be discharged, directed to BIO for 24 h observation, or immediately admitted to the neurologic ward for further diagnostic procedures and therapies. Patients discharged from either the ED or BIO, but requiring differential diagnosis or advancement to a second level of therapeutic program management should be referred to the Day Service.

Within a hospital organization, the presence of a BIO within the ED may allow the neurologist to monitor the clinical course of a patient under observation and encourage full recovery without formal ward admission and without taking unreasonable risks derived from a hasty discharge. Similarly, advanced outpatient services, such as Day Services, may ensure effective and rapid diagnostic assessment in patients who require further and timely examinations. Although this algorithm is centered on resources based on current Italian laws, this model could easily be adopted by worldwide healthcare systems. The diffusion of teleconsultation devices worldwide may enable fast and effective management of neurological emergencies, even in rural and mountainous basic healthcare facilities, isolated from either regional hospitals (16). Organizational models enabling expedited access to neurological clinics while relieving overwhelmed EDs have already been designed and have produced positive outcomes (17). In this model, the Day Service was conceived and worked effectively. This organizational model aims to address patient health needs while simultaneously improving the cost/effectiveness ratio. Incorporating both hospitals and territorial healthcare facilities, through teleconsultation and telemonitoring, is critical for effective performance of the model.

The use of the BIO ward or neurologic Day Services for each outpatient LEA have been recently activated in the ED and neurological ward in our hospital in Barletta, and are expected to enable favorable outcomes for the economic sustainability of high-quality healthcare.

## Author contributions

MG conceived the work, collected the data, performed statistical analysis, draw all figures, wrote the initial draft, and corrected the final one. PDF helped in conceiving the work, discussed statistical analysis and figures, and performed critical review of the initial draft. All authors contributed to the article and approved the submitted version.

## Footnotes

^1^“Gazzetta Ufficiale” n 33: February 08, 2002.

^2^The National Recovery and Resilience Plan (NRRP). Decreto Legislativo 77/2022.
